# The protocol and design of a randomised controlled study on training of attention within the first year after acquired brain injury

**DOI:** 10.1186/1471-2377-14-102

**Published:** 2014-05-08

**Authors:** Aniko Bartfai, Gabriela Markovic, Kristina Sargenius Landahl, Marie-Louise Schult

**Affiliations:** 1 Karolinska Institutet, Department of Clinical Sciences Danderyd Hospital, Division of Rehabilitation, Department of Rehabilitation Medicine, Danderyd University Hospital, Stockholm 182 88, Sweden

**Keywords:** Brain injury, Attention, Statistical process control, Cognitive rehabilitation, Early rehabilitation, Work return, Health economics

## Abstract

**Background:**

To describe the design of the study aiming to examine intensive targeted cognitive rehabilitation of attention in the acute (<4 months) and subacute rehabilitation phases (4–12 months) after acquired brain injury and to evaluate the effects on function, activity and participation (return to work).

**Methods/Design:**

Within a prospective, randomised, controlled study 120 consecutive patients with stroke or traumatic brain injury were randomised to 20 hours of intensive attention training by Attention Process Training or by standard, activity based training. Progress was evaluated by Statistical Process Control and by pre and post measurement of functional and activity levels. Return to work was also evaluated in the post-acute phase. Primary endpoints were the changes in the attention measure, Paced Auditory Serial Addition Test and changes in work ability. Secondary endpoints included measurement of cognitive functions, activity and work return. There were 3, 6 and 12-month follow ups focussing on health economics.

**Discussion:**

The study will provide information on rehabilitation of attention in the early phases after ABI; effects on function, activity and return to work. Further, the application of Statistical Process Control might enable closer investigation of the cognitive changes after acquired brain injury and demonstrate the usefulness of process measures in rehabilitation. The study was registered at ClinicalTrials.gov Protocol.

**Trial registration:**

NCT02091453, registered: 19 March 2014.

## Background

Cognitive changes after acquired brain injury (ABI) constitute a major challenge both for the ABI survivor and society. Cardinal symptoms are memory impairment, attention deficit, executive dysfunction and fatigue observed at level of function as well as at level of activity and participation. The two largest diagnostic groups with ABI are traumatic brain injury (TBI) [1] and stroke [2]. There are large individual and social gains to be achieved on minimising the short- and long-term effects through rehabilitation.

Attention, i.e. the allocation of processing resources, has been found to be one of the cognitive functions that is successfully improved through systematic training after ABI [3]. Attention Process Training (APT) was found to be one of the successful restorative methods in adults [3,4] during the late, chronic phase after ABI. Meta analytic reviews [5] found an effect size of 35–38% for domain-specific training.

In the acute and subacute phases, within one year after ABI, results are more conflicting. Novack and his coworkers [6] provided patients in the acute phase with 10 hours of unstructured and structured attention training, based on the Sohlberg-Mateer hierarchical model, and found that the observed differences were most likely to reflect spontaneous recovery. Ponsford and Kinsella [7] administered 15 hours of computer-based attention training for subjects within the first year after moderate to severe head injury but could not report treatment effects on having controlled for the effect of spontaneous recovery. Sturm and Wilmes [8] on the other hand found significant treatment effects in a stroke group after seven hours of computer-based training for a number of attention functions but without generalisation to other cognitive functions. Cicerone and co-workers [9] concluded that there was insufficient evidence to distinguish the effects of spontaneous recovery from cognitive training in the acute phase after TBI or stroke.

Brain injury rehabilitation comprises of interventions targeting changes at behavioural level working with compensatory behaviour, and at restorative level in improving the lost function itself [10]. Outcome can be evaluated by behavioural measures such as psychometrical testing or observation and evaluation of activity and participation. Assessment and evaluation of treatment progress has been limited so far by methodological issues, the selection of assessment instruments and their sensitivity to changes. One of the methodological issues when measuring behavioural changes has been the emphasis on endpoint measures, rather than a detailed analysis of behaviour [11].

Rehabilitation involves time and resource-consuming interventions, which might need closer monitoring. Statistical Process Control (SPC) is a method that considers the variability in a process to better understand whether the intervention has a desired impact [12]. This method has been increasingly applied in health care as a quality-monitoring tool [13]. However, in the field of brain injury rehabilitation, only one article has been published [14]. In rehabilitation research, detailed analyses of the process of recovery have been used in single case studies [8,15]. Process analyses to describe patterns of recovery and restitution in brain injury rehabilitation programmes at group level have, to our knowledge, not been applied.

Studies concerning effects of attention training on everyday functional activities are more limited due to methodological difficulties [16,17]. However, Björkdahl [18] recently reported clinically relevant improvements at a functional level in patients receiving computerised working memory training.

The beneficial effects of vocational rehabilitation have been investigated in a number of studies [19,20]. There is, however a lack of studies examining the effectiveness of attention training on work return, although several studies have found that cognitive impairment has a far greater effect on work return, than physical disabilities [21,22].

In a recent multicentre study Oddy, M & da Silva Ramos, [23] found significant economical gains for patients provided with neurorehabilitation. The results also indicated that neurorehabilitation within the first year after ABI results in higher economical gains than when implemented later on. The specific effects of cognitive training from an economic perspective have not yet been examined.

Intensive targeted rehabilitation of specific cognitive functions is an emerging area with good potential for individuals after ABI to improve performance, decrease activity limitations and thus potential gains for the individual and society. Deeper knowledge about the use of the methods, such as timing and extent of training, training of generalisation, effects on everyday activities and working capacity is limited. The present study was aimed at some of those specific aspects also using statistical process control (SPC) [12] methodology. The rationale and design of the study is presented below.

## Methods/Design

The primary objectives of the study were: 1. In the acute stage, to evaluate the effectiveness of APT measured by the changes in performance in the attention measure, Paced Auditory Serial Addition Test, PASAT [24] evaluated by SPC [12] 2. In the postacute stage, to evaluate and compare effects of APT vs. standard rehabilitation on daily activities and return to work three months and one year after participating in a rehabilitation programme with regard to resource utilisation and health economics.

Several secondary objectives were also established as defined below:

•Evaluate the effects of APT training compared to standard rehabilitation of attention on other cognitive function

•Evaluate the effects of APT training compared to standard rehabilitation on activity limitations

•Evaluate the effects of APT training compared to standard rehabilitation on participation e.g. work return, self assessed work ability and individual skills during work performance at the end of rehabilitation program and 3 months after rehabilitation

•Identify cognitive predictors for successful work return, self assessed work ability and individual skills during work performance

•Evaluate the effects of APT training compared to standard rehabilitation at 6 months follow up on other cognitive functions

•Evaluate and compare effects of APT vs. standard rehabilitation between acute and postacute patients

•on other cognitive functions

•on activity limitations

•Evaluate and compare effects of APT vs. standard rehabilitation on work return one year after participating in rehabilitation program, on resource utilization and economic effects

**Figure 1:**
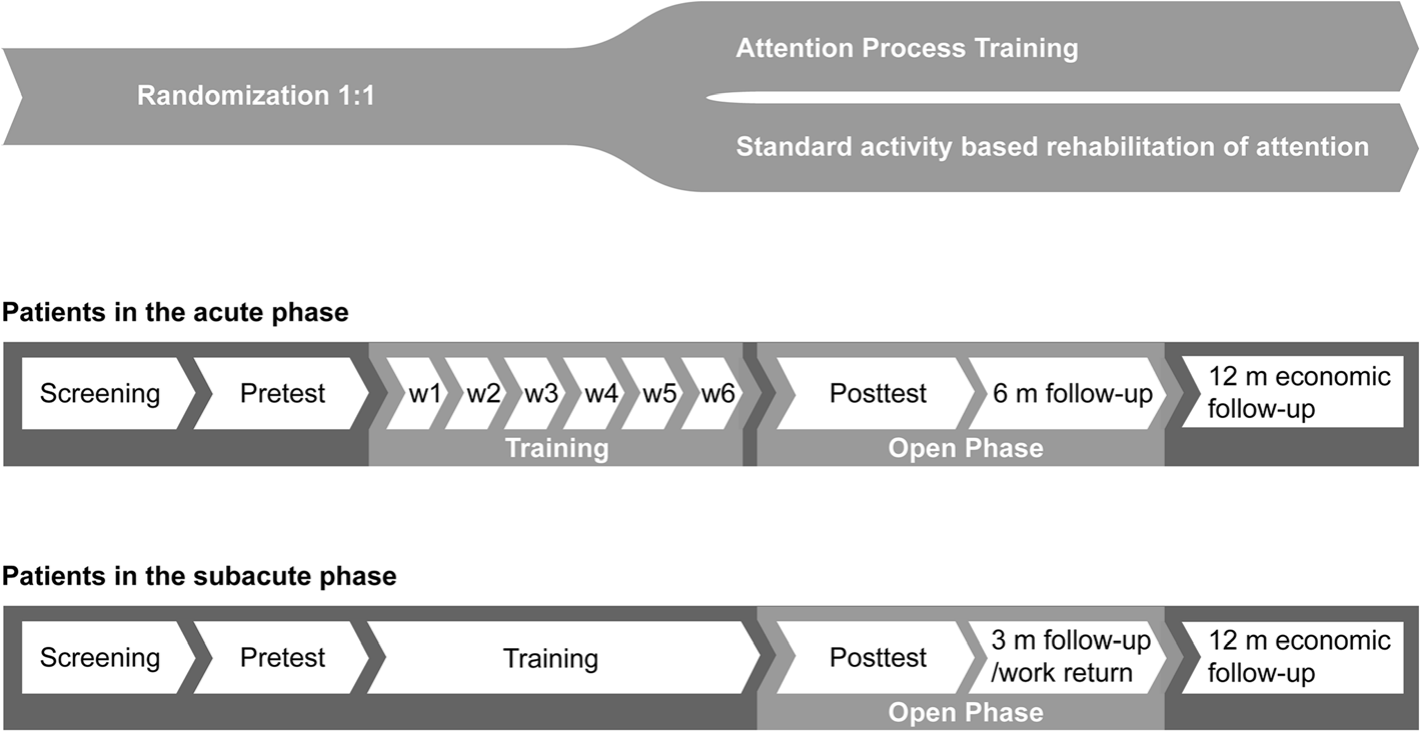
Study plan.

The study was conducted in a specialised rehabilitation clinic. Several in- and outpatient units on two sites were involved. It was a randomised, controlled (http://www.consort-statement.org/?o=1011) study with an open extension and a follow up at three and six months and one year. Due to the nature of the rehabilitation procedures, neither patients nor rehabilitation professionals were blinded as to the nature of the intervention, but different professionals conducted assessments and training. The study was designed to reflect normal clinical practice while allowing comparison between two rehabilitation approaches. Patients in the acute phase (<4 months) were recruited from both in- and outpatient units. For those, ready to be discharged from inpatient wards, the training was continued in the outpatient setting. Subacute patients (4–12 months) participated in the study as part of their outpatient rehabilitation (Figure 1). The study was approved by the regional ethics committee, Karolinska Institutet.

### Study population

The participants comprised a consecutive series of patients with mild to moderate stroke or TBI. Inclusion criteria were deficits in attention defined by the APT test [4], scores on the lower average and above for reasoning skills and abstract thinking, age range; 18–60 years and a good understanding of the Swedish language. Exclusion criteria were moderate to severe aphasia, ongoing psychiatric illness, a history of severe somatic disorder causing anoxic periods, ongoing substance abuse and severe pain. Patients with Hospital Anxiety and Depression Scale (HADS) [25] scores = > 10 were offered antidepressant treatment and were included in the study three weeks after pharmacological treatment had been initiated. Further, exclusion was also based on severe memory disorders, neglect, visual field defects and motor disability interfering with participation.

### Study schedule

All patients participated in a comprehensive interdisciplinary brain injury rehabilitation programme. After baseline assessment, the participants received 20 hours of attention training, at least three days a week, for a period of 5–6 weeks. They were randomly assigned to one of the two intervention programmes. One group of participants underwent intensive area-specific cognitive training with the APT [4]. The other group of participants received activity-based attention training provided by the occupational therapist. Evaluation of treatment effects was monitored by pre and post assessment and by repeated assessment by the primary outcome measure at baseline, after every third hour of intervention and post intervention for patients in the acute stage. Subacute patients were assessed by pre and post measurements. The open-label extension of the study comprised of client-centred standard interdisciplinary rehabilitation. Discharge was based on clinical decisions and the participants returned to the clinic for follow-up assessments (Table 1).

**Table 1:** Study schedule and study measures

		**Study measures**	**Inclusion exclusion**	**Pretest**	**Posttest**	**Weekly assessment**	**Follow-up 3 months**	**Follow-up 6 months**
**Inclusion/Exclusion criteria**		APT Test	x					
		Matrices (WAIS-III)	x					
		Alberts test	x					
		Barthel ADL-index	x					
		RBMT	x	x	x			
**Primary outcome measures**		PASAT		x	x	x*		x
		WAI					x	
**Secondary outcome measures**	**Function**	Digit Span (WAIS-III)		x	x			x
		Color-Word (D-KEFS)		x	x			x
		Block Span (WAIS-III-NI)		x	x			x
		Ruff 2 & 7		x	x			x
		Trails Test (D-KEFS)		x	x			x
		Letter-number Sequencing (WAIS-III)		x	x			x
		RAVLT		x	x			x
		Tower Test (D-KEFS)			x			
		HADS		x	x			x
	**Activity**	CFQ		x	x			x
		DEX (BADS)		x	x			x
		RSAB		x	x			
		COMP		x	x		x**	
	**Participation and work return**	WAI		x**	x**		x**	
		Work ability screening		x**	x**		x**	
		AWP		x**	x**		x**	
		QWT		x**	x**		x**	

*Acute phase only.

**Subacute phase only.

### Training methods

APT [4] was used as intensive area-specific cognitive training. The programme provides a theoretically based, individualised, highly structured intervention of organised assignments at four attention levels: sustained, selective, divided and alternating attention. Progress is based on the intensity of training, on continuous feedback promoting motivation and on metacognitive training. The APT also includes education in acquired attention deficits and training for generalisation, enabling the transfer of treatment tasks and techniques to self-selected cognitive problems in everyday situations. Each session took 45–90 minutes to perform, working with material from APTs I and II [26]. The APT-test was used to determine the level of difficulty for the attention training with the APT and to measure improvement in performance.

Attention training in activities included standard occupational training within an interdisciplinary rehabilitation programme. The programme consisted of a) training and the use of compensatory strategies in attention-demanding activities of daily living b) performing independent work with attention-demanding tasks at individual level c) training using computerised tasks, not specifically designed for attention remediation and d) group activities. Types of training and time devoted to a specific training procedure were individually registered.

### Training of trainers and evaluators

APT training was administered by three trainers: one neuropsychologist and two occupational therapists. During the first year of data collection ongoing training was discussed on a weekly basis. Attention training in activity was supervised by one of the investigators. The neuropsychologist administered cognitive assessment, and the occupational therapists administered the activity and participation measurements. The evaluators received individual training for each instrument to assure interrater reliability and conformance to data collection. All occupational therapists working in the subacute phase attended a course in Assessment of Work Performance AWP [27] and Assessment of Work characteristics (AWC) [28].

### Assessments

#### Inclusion/exclusion measures

Inclusion measures were the APT-test [4] and Matrix reasoning from the WAIS-III [29]. Cut-off scores were 70% or less on at least two of the five subtests on the APT-test and standard scores of seven and above on Matrix Reasoning.

Exclusion measures were Barthel ADL-index [30], 50 or less; Alberts test/Line crossing [29] with a cut off score of (<=2); and a profile score of seven or less for The Rivermead Behavioural Memory Test (RBMT) [31].

#### Primary outcome measures

For the acute phase: The Paced Auditory Serial Addition test, (PASAT) is presumed to measure working memory speed of information processing and sustained and divided attention [24]. Versions A (isi 2,4 sec) and C (isi 1,8 sec) were administered. Scores are the number of correct responses. Higher scores indicate better performance.

For the subacute phase the primary outcome measurement was the Work Ability Index (WAI) [32]. Higher scores indicate better performance.

#### Secondary outcome measures

Secondary outcome measures and study schedule are presented in Table 1.

### Functional measures

Further measures of attention were the Digit Span task [29]. Forward repetition of digits is considered to assess verbal attention span and backward repetition is considered to assess working memory [29]. The scores obtained were the total sum of forward and backward, and longest forward span.

The Block Span [29] is a nonverbal subtest corresponding to the Digit Span. The scores obtained were the total sums, forward and backward, respectively.

The Ruff 2&7 Selective Attention Test measures visual automatic detection speed and accuracy and controlled search speed and accuracy [33]. Scoring was based on the manual.

The Letter-Number Sequencing task [29] was developed to increase sensitivity to attention deficits. The score is the sum of correctly repeated series. Higher scores indicate better performance.

The Trail Making Test (TMT) used in the present study is a part of the Delis-Kaplan Executive Function Scale (D-KEFS) [34], measuring visual scanning, graphomotor speed and mental flexibility. Time scores for sections 2, 3 and 4 and number of errors for section 4 were reported. Lower scores indicate better performance.

The Stroop Test paradigm is used in the Color-Word Interference Test (D-KEFS) [34] consisting of four parts, 1. Color naming, 2. Color reading, 3. Inhibition, 4. Inhibition and flexibility. Scores were the time required for completion and number of errors. Lower scores indicate better performance.

The Tower Test is also a part of the D-KEFS battery [34] requiring planning, working memory, visuospatial memory and response inhibition [29]. The test is administered only once due to learning effects, thus allowing only group comparisons. In addition to the scoring according to the manual, the number of correct solutions, number of moves and number of broken rules were also reported.

The Rey Auditory Verbal Learning Test (RAVLT) [29] has been used to evaluate different aspects of memory function. Scores comprised the number of correctly repeated words on the first, fifth and interference trials, the total number of repeated words, the number of words at immediate and delayed recall. Confabulations were also tallied. Higher scores indicate better performance in all measures except for confabulations.

Depression and anxiety were assessed by the Hospital Anxiety and Depression Scale (HADS). A score of < 7 on the depression subscale indicated no signs of depression, a score of 8–10 indicated mild signs of depression and > 10 points indicated that the participant suffers from depression [25].

### Activity measures

The Cognitive Failure Questionnaire (CFQ) [35] is a self-report instrument consisting of 25 questions and aimed at capturing consequences of cognitive problems in daily living. Scores range from 0–4, maximum score is 100. High scores imply frequent cognitive problems.

The Rating Scale of Attentional Behavior (RSAB) [36] assessed the impact of attentional impairment on the everyday behaviour of the patient. Scoring was performed jointly by the patient’s occupational therapist and physiotherapist. The maximum score is 56 and lower scores imply better performance.

The Dysexecutive symptom questionnaire (DEX) is part of the Behavioural Assessment of Dysexecutive Syndrome (BADS) [37] which captures consequences of poor planning and reasoning in everyday living. In the present study only the patient form was used. Scores range from 0–4, maximum score is 80. Higher scores indicate more dysexecutive symptoms.

Occupational Performance was measured by the Canadian Occupational Performance Measure (COPM) [38]. Two scores were obtained, one score for occupational performance and one for satisfaction with performance in everyday activities. Higher scores reflected better performance and satisfaction. According to the manual, a change of two or more points on the COPM score is considered clinically relevant [38].

### Participation measures – work-return

#### The Work Ability Index (WAI)

The Work Ability Index (WAI) was used [32] to assess self-rated work ability. The WAI is a self-report questionnaire containing 10 questions. A total score can be calculated by weighting all items to an index score, which can be grouped into four classes 1. “poor” work ability, score 7–27 (need to restore work ability) 2. “moderate” work ability, score 28–36 (need to improve work ability), 3: “good” work ability, score of 37–43 (need to support work ability) 4: “excellent” work ability, score of 44–49 (need to maintain work ability). The total WAI score as well as results for the separate questions are presented.

#### Assessment of Work Performance (AWP)

The Assessment of Work Performance (AWP 1.1) can be used to assess the skills of clients with various work-related problems during their work performance – how efficiently and appropriately the client performs a work task. Skills are assessed in three domains: motor skills, process skills, and communication and interaction skills [27]. These skills are numerically and individually rated on a four-point Likert-type scale. Irrelevant, or impossible to assess items are marked separately.

#### Work ability screening questionnaire

A questionnaire constructed by the authors including questions about education, current profession, work situation, current work ability, approach to future work ability and return to work questions.

#### Work ability questionnaire

The current work ability and work situation is followed over time using this questionnaire constructed by the authors. Responses are recorded at the end of the rehabilitation in the subacute phase and after an additional three months.

### Measurement of adverse effects

Adverse effects were registered in field notes and standard hospital journals. Examples of adverse effects were: fatigue preventing participation in APT training, negative effects of the APT treatment, such as consequences of fatigue on following treatments, sudden emergence of exclusion criteria, etc.

### Resource utilisation and economic evaluation

Data for work capacity/sick leave before, and 12 months after ABI were obtained from the Registry for the Swedish Social Insurance Agency. For data regarding utilisation of health services related to ABI and medical and physical interventions, the participants were contacted by phone 12 months after injury. Resource utilisation was defined as the use of health care and social services associated with ABI. Costs of resources were calculated from a societal perspective, i.e. costs within the health-care system, transport, and caregiver’s time and for sick leave. Patients’ and caregivers’ lost productivity was calculated based on productivity data from before ABI [39].

### Study endpoints

#### Primary endpoint for the acute phase

The primary endpoint was performance in the attention measure PASAT after 20 hours of APT training expressed as the number of correct responses. For comparisons with other functional and activity measures different scores were developed.

#### Primary endpoint for the subacute phase

The primary endpoint was the score in the WAI measure expressed as the degree of subjective work ability. The difference scores (dS1 = Score_after training_ – score_before training_; dS2 = Score_at follow up_ – score_before training_; dS3 = Score_at follow up_ – score_after training_) will be used.

#### Secondary endpoints

A number of secondary endpoints involving measures on functional and activity levels, after training and at follow up, work-return and patient-reported outcome variables were also evaluated during the study as defined below

Outcome after the training

•on functional level

•on other test of attention control

•on test requiring motor control

•on test requiring executive control

•on declarative memory

•on activity level

Outcome at follow-up

•on functional level

•on activity level

•in participation (work return)

Economic outcome

### Statistical design and analysis

#### Sample size calculations

Sample size calculations were done in IBM SPSS Sample Power. The sample size calculation for the primary endpoint was based upon the estimate of 1 SD improvement after 20 hours of attention training with APT or activity-based attention training. Setting an alpha at 0.05, with a power of 85%, a sample size of 19 completed data sets was needed to detect a statistically significant difference between treatment arms. Assuming a dropout rate of 25% requires the inclusion of 25 patients. Sample size calculation for secondary endpoint goals on functional level was based upon the assumption of a clinically relevant change of 1 SD in the Ruff 2&7 test. For this effect size, a sample size of 30 complete data sets in each treatment arm, and alpha 0.0050, 2-tailed, yields a power of 0.888. Sample size calculation on activity level was based upon the assumption of a clinically relevant (2-point) change in the COPM performance measure. For this effect size, a sample size of 30 complete data sets in each treatment arm, and alpha 0.0050, 2-tailed, yields a power of 0.988. Thus the maximum number of patients to be enrolled was 120.

#### Process analyses

The primary outcome measure was analysed by using statistical process control (SPC). Sigma Zone SPC XL, was used to explore statistical control limits and variability in improvement assuming that data plots appearing within the control limit indicate a process in stable statistical control and variations are due to chance variations, day-to-day variability in behaviour etc. Data were presented on control charts, including three additional lines; the centre line (usually based on the mean) and an upper (UCL) and lower control limit (LCL) set at ±3 standard deviations from the mean respectively [40]. Data points outside those control limits are considered to be related to special causes of variation, such as effects of treatment. Patterns were analysed according to run analysis: any one point that falls outside the control limits (i.e. above the UCL or below the LCL) (one), seven or more consecutive points all above or below the centre line (the mean) (a run) and seven or more consecutive points moving up or down bisecting the centre line (a trend) [12]. The Minimal Clinically Important Difference (MCID) in the primary outcome variable, PASAT was estimated and expressed as the minimum change of the PASAT-diff score that could be considered clinically relevant.

#### Data analysis

Data were stored and analysed in IBM SPSS Statistics 20. Data were checked for skewness and kurtosis. Parametric methods, Student’s t-test and Pearson correlation were used for normally distributed variables on interval level. When comparing the two treatment groups, t-tests for independent samples were used and for comparison between pre- post and follow up measures t-tests for dependent samples and analysis of variance were used. Skewed or ordinal data were analysed by non-parametric methods: Mann–Whitney U-test for comparison between the treatment groups; Wilcoxon matched pairs test, Kruskal-Wallis analysis of variance for comparison of pre- post and follow up measures and Spearman’s rank correlation test. For post-hoc comparisons we used the Mann–Whitney U-test. Fisher’s exact test was used for comparison between dichotomized variables. Bonferroni corrections were applied to correct for false positives due to the number of analyses. The possible effects of randomisation bias were investigated using a linear mixed-model analysis including one within-group factor treatment and one between-group factor. Cluster analysis was used for the primary outcome measure to explore and identify patterns of cognitive recovery in the acute phase.

Two-tailed p-values were used with a critical significance level of 0.05.

### Health economics

Resource utilisation was calculated for each treatment arm for participants in both acute and subacute phases. Costs were estimated for each participant summarised according to treatment group and rehabilitation phase (acute, subacute). Descriptive statistics were calculated for resource utilisation and costs over 12 months and an analysis of variance (ANOVA) was applied to test the differences between treatment arms.

For health economics the SF-6D (6 dimensions) and EQ-5D will be used. We will conduct health economic evaluations from a societal perspective and include production, cost-benefit analysis and quality-adjusted life years QUALYs, as endpoints. QUALYs are used since the effects are measured by the patients themselves, and the measure may therefore be considered to reflect preferences. An analysis comparing the two alternative treatments results in a cost-effectiveness ratio expressed as cost per QUALY, i.e. the additional cost of the (new) more expensive treatment is divided by the difference in effect (number-generated QUALYs) between the new and the old treatment/rehabilitation.

## Discussion

Patients in this study were consecutively recruited and carefully randomised, but the obvious differences in the rehabilitation interventions counteracted the possibility of withholding information about group membership (intervention/control). Training and pre- and post measurements were administered by different rehabilitation professionals, and those performing the measurements were not aware of the patients’ status in the project, but probable placebo effects cannot be excluded. Patients with ABI after stroke or TBI were selected on the basis of symptomatology, but not aetiology and the statistical power in the present sample size was insufficient for subgroup analyses according the aetiology of ABI. Further data collection would be required to achieve adequate samples.

Although this study was designed to capture information within standard rehabilitation, participation in the study included more frequent contacts with rehabilitation professionals than generally occur in a clinical setting. Pre- and posttest sessions and follow-up assessments implied a higher frequency of contact than standard rehabilitation care. Repeated testing with the primary outcome measure, PASAT, in the acute phase can be regarded as a separate form of training in divided attention and parallel processing. This training effect, albeit for both treatment arms, might have incremented the effectiveness of rehabilitation as opposed to standard care and impacted outcome measures. There is also the question of generalisability of results. Patient inclusion was based on strict criteria for research purposes, and the representativity of the sample has to be determined by careful comparison between all patients admitted to the clinic with the same diagnosis and the present sample.

It is anticipated that the study will provide a wealth of information regarding cognitive rehabilitation of attention in the early (<4 months) and subacute phase (4 months-12 months) after acquired brain injury; effects on activity level and on work re-entry. Resource utilisation and costs will provide additional information about the treatment efficacy. Furthermore, the application of a process measure might allow deeper insights into the process of spontaneous recovery after brain injury and rehabilitation processes in general.

## Abbreviations

ABI: Acquired brain injury; ANOVA: Analysis of variance; APT: Attention Process Training; AWP: Assessment of Work Performance; AWC: Assessment of Work Characteristics; BADS: Behavioural Assessment of Dysexecutive Syndrome; CFQ: Cognitive Failure Questionnaire; COPM: Canadian Occupational Performance Measure; DEX: Dysexecutive symptom questionnaire; D-KEFS: Delis-Kaplan Executive Function Scale; HADS: Hospital Anxiety and Depression Scale; LCL: Lower control limit; MCID: Minimal Clinically Important Difference; PASAT: Paced Auditory Serial Addition Test; QUALYs: Quality-adjusted life years; RSAB: Rating Scale of Attentional Behavior; RAVLT: Rey Auditory Verbal Learning Test; RBMT: Rivermead Behavioural Memory Test; SPC: Statistical process control; TBI: Traumatic brain injury; TMT: Trail Making Test; UCL: Upper control limit; WAI: Work ability index.

## Competing interest

The authors declare that they have no conflict of interests.

## Authors’ contributions

AB is the head of the research group and main supervisor for GM and KSL. She conceived of the study, and participated in its design and coordination and helped to draft the manuscript from the point of view of neuropsychology and assessment of cognitive training. MLS contributed to conception, design of the study and helped to draft the manuscript with particular emphasis on activity, participation and health economy measures. GM contributed to the conception and design of the study from a neuropsychological perspective and is responsible for acquisition of data in the acute phase. She is also responsible for the application of different statistical methods in the evaluation process, particularly regarding neuropsychological data. She has also participated in the draft the manuscript. KSL contributed to conception and design of the study. She is responsible for establishing controlled treatment conditions for the generalisation of APT treatment and for comparable multiprofessional rehabilitation. She is also responsible for acquisition of data in the subacute phase with a particular emphasis on measures of work return. She has participated in the draft the manuscript. All authors read and approved the final manuscript.

## Pre-publication history

The pre-publication history for this paper can be accessed here:

http://www.biomedcentral.com/1471-2377/14/102/prepub
